# Evaluation of Diagnoses, Discharge Rates, Follow Up Frequency, and Non-Attendance of South Caerphilly Community Mental Health Team Outpatient Psychiatric Clinics

**DOI:** 10.1192/bjo.2025.10470

**Published:** 2025-06-20

**Authors:** Mohamed Bader, Rahul Bansal

**Affiliations:** Aneurin Bevan UHB, Caerphilly, United Kingdom

## Abstract

**Aims:** Evaluate the frequency of mental disorders patients present with in different sites in SCCMHT to inform quality improvement to better match patient needs. To assess the non-attendance rate in various sites for future projects to explore factors associated with patient non-attendance. To quantify outcomes following patient reviews to explore discharge/follow up frequency.

**Methods:** The service evaluation was done retrospectively for three-month period from September to November, 2024, by collecting data from WCCIS database. Patient WCCIS identifiers were only stored in the spreadsheet used. Descriptive statistics were used to identify the most common diagnoses, outcomes, and non-attendance rates through various methods of stratifying the data. The data was compared when split between consultant clinics vs speciality doctor, Risca Health Centre Base vs Mill Road Base, and between urgent and non-urgent clinics.

**Results:** Mill Road and Risca OPA clinic had 172 and 76 appointments attended respectively over three months. Mill Road OPA had higher rates of non-attendance (26.18%) compared with Risca OPA (21.65%). Patients in Risca clinic are booked in for less frequent follow ups (72% with 4–6 months of follow up). A substantial percentage of patients being seen urgently are subsequently discharged from the CMHT (29%). This includes urgent patients seen in the Home Treatment Team Clinic, which often uses urgent consultant reviews to support discharge of patients. DNA rates are similar between consultant and speciality doctor clinic (23.2% and 25.85% respectively). DNA rates are approximately 1/4 across total clinics (24.84%). Despite a separate ADHD service and a supervised Physician’s Associate Clinic for ADHD separately, ADHD reviews still account for a considerable amount of outpatient clinic time (32 among 248 consultations). Similarly, PTSD/CPTSD and Borderline Personality Disorder account for a large number of urgent reviews (13 among 34 patients consulted).

**Conclusion:** DNA rates would require further assessment. They have not been included in analyses of diagnoses; a further exploration of the data is indicated to identify factors/diagnoses associated with non-attendance. A high prevalence of patients requiring urgent review are patients with borderline personality disorder and PTSD/CPTSD indicating Trauma Informed Approaches especially in crisis are required. Demands for ADHD assessments/follow ups remain high despite the presence of a ADHD service and Physician’s Associate Clinic (review of stable shared care patients). Patients remain under the CMHT even if they have no additional CMHT needs outside of Shared Care ADHD reviews, highlighting ongoing resource demand on the CMHT.

